# Supplementary material for: Comparison of Selected Immune and Hematological Parameters and Their Impact on Survival in Patients with HPV-Related and HPV-Unrelated Oropharyngeal Cancer
Source: Cancers (Basel). 2021 Jun 29;13(13):3256. doi: 10.3390/cancers13133256 (PMC8268778; doi:10.3390/cancers13133256)
Supplement: Supplementary file 1 [file cancers-13-03256-s001.zip › cancers-1255922 suppl.pdf]

cancers

MDPI

Article

Comparison of Selected Immune and Hematological Parameters and Their Impact on Survival in Patients with HPV-Related and HPV- not Related Oropharyngeal Cancer

Adam Brewczyński [1], Beata Jabłońska [2], Agnieszka Maria Mazurek [3], Jolanta Mrochem-Kwarciak [4], Sławomir Mrowiec [2], Mirosław Śnietura [5], Marek Kentnowski [1], Zofia Kołosza [6] , Krzysztof Składowski [1] and Tomasz Rutkowski [1]

[1] I Radiation and Clinical Oncology Department of Maria Sklodowska-Curie National Research Institute of Oncology, Gliwice Branch, Poland
[2] Department of Digestive Tract Surgery, Medical University of Silesia, Katowice, Poland
[3] Centre for Translational Research and Molecular Biology of Cancer of Maria Sklodowska-Curie National Research Institute of Oncology, Gliwice Branch, Poland
[4] The Analytics and Clinical Biochemistry Department of Maria Sklodowska-Curie National Research Institute of Oncology, Gliwice Branch, Poland
[5] Tumor Pathology Department of Maria Sklodowska-Curie National Research Institute of Oncology, Gliwice Branch, Poland
[6] Department of Biostatistics and Bioinformatics of Maria Sklodowska-Curie National Research Institute of Oncology, Gliwice Branch, Poland
* Correspondence: author: bjablonska@poczta.onet.pl

Supplementary

Table S1. Pre-treatment and post-treatment laboratory (peripheral blood morphology parameters) results.

	**HPV(-)**	**HPV(+)**	**p**
Hb 0 [g/dl]	14.05±1.51	13.88±1.51	0.530
Hb 1 [g/dl]	12.03±1.54	12.03±1.54	0.331
Hb 01 [g/dl]	1.75±1.46	1.85±1.58	0.717
RetHb 0 [/mm^3]	34.04±3.36	34.21±2.61	0.762
RetHb 1 [/mm^3]	33.50±3.38	35.01±1.43	0.031
RetHb 01 [/mm^3]	0.48±4.31	-0.73±2.91	0.078
RBC 0 [/mm^3]	4.51±0.53	4.61±0.54	0.274
RBC 1 [/mm^3]	4.00±0.55	3.94±0.57	0.531
RBC 01 [/mm^3]	0.51±0.52	0.68±0.54	0.085
Ret 0 [/mm^3]	51.97±24.96	60.56±22.73	0.052
Ret 1 [/mm^3]	51.20±25.29	48.62±23.88	0.574
Ret 01 [/mm^3]	0.89±29.78	12.30±30.51	0.044
WBC 0 [/mm^3]	7.15±2.03	6.45±1.91	0.048
WBC 1 [/mm^3]	5.12±2.29	4.35±2.25	0.059
WBC 01 [/mm^3]	2.00±2.87	2.10±2.70	0.846
CLC 0 [/mm^3]	1.91±0.71	1.89±0.73	0.842
CLC 1 [/mm^3]	0.71±0.44	0.52±0.24	0.004
CLC 01 [/mm^3]	1.20±0.72	1.37±0.69	0.174
CNCC 0 [/mm^3]	4.34±1.86	3.77±1.49	0.064
CNCC 1 [/mm^3]	3.67±1.84	3.24±2.10	0.226
CNCC 01 [/mm^3]	0.66±2.55	0.54±2.20	0.779
CMC 0 [/mm^3]	0.64±0.25	0.56±0.24	0.057
CMC 1 [/mm^3]	0.61±0.32	0.48±0.20	0.012
CMC 1 [/mm^3]	0.04±0.33	0.08±0.28	0.502
PLT 0 [/mm^3]	256.93±77.16	236.76±57.94	0.103
PLT 1 [/mm^3]	250.31±118.11	208.15±65.18	0.016
PLT 01 [/mm^3]	7.25±119.63	28.61±60.37	0.218

Values are presented as means ± standard deviations.

0, before treatment, 1, after treatment; 01, difference; Hb, hemoglobin level; RetHb, ret-hemoglobin level; RBC, red blood cells; Ret, reticulocyte count; WBC, white blood cells count; CLC, circulating lymphocyte count; CNC, circulating neutrophil count; CMC, circulating monocyte count; PLT, platelet cells count.

Table S2. Pre-treatment and post-treatment immune ratios.

	**HPV(-)**	**HPV(+)**	**p**
NLR 0	2.71±2.14	2.31±1.35	0.216
NLR 1	6.66±4.95	7.59±6.38	0.355
NLR 01	-3.94±5.28	-5.29±6.05	0.186
MLR 0	0.36±0.16	0.31±0.15	0.053
MLR 1	1.00±0.54	1.10±0.70	0.385
MLR 01	-0.64±0.53	-0.79±0.65	0.154
PLR 0	152.25±73.47	145.09±77.14	0.593
PLR 1	436.38±249.77	486.40±251.00	0.265
PLR 01	-283.25±228.41	-341,31±226.87	0.156
SII 0	707.36±586.07	553.31±369.82	0.084
SII 1	1638.77±1406.1	1471.13±1053.4	0.453
SII 01	-929.62±1430.54	-917.81±948.99	0.957

Values are presented as means ± standard deviations.

0, before treatment, 1 after treatment; 01, difference; NLR, neutrophil/lymphocyte ratio (NLR); LMR, lymphocyte/monocyte ratio; PLR, platelet/lymphocyte ratio; SII, systemic immune inflammation index.

Table S3. Comparison of low and high neutrophil/lymphocyte ratio (NLR) groups according to selected clinicopathological factors in HPV(-) and HPV(+) patients.

	**HPV-**			**HPV+**		
	**NLR < 2.71**	**NLR ≥ 2.71**	**p**	**NLR < 2.31**	**NLR ≥ 2.31**	**p**
Age [years]	61.2±6.71 (50-79)	60.0±9.01 (37-74)	0.937	60.7±10.85 (30-80)	59.8±7.46 (45-76)	0.520
Gender						0.422
M	34 (73.9%)	17 (77.3%)	1.00	21 (56.8%)	15 (68.2%)	
F	12 (26.1%)	5 (22.7%)		16 (43.2%)	7 (31.8%)	
General location			**0.031**			0.748
Tonsil	34 (73.9%)	10 (45.5%)		30 (81.1%)	17 (77.3%)	
Others	12 (26.1%)	12 (54.5%)		7 (18.9%)	5 (22.7%)	
Detailed location			**0.039**			0.872
1. tonsil	34 (73.9%)	10 (45.5%)		30 (81.1%)	17 (77.3%)	
2. palate	6 (13.0%)	4 (18.2%)		0 (0.0%)	0 (0.0%)	
3. root of the tongue	5 (10.9%)	8 (36.4%)		5 (13.5%)	4 (18.2%)	
4. other oropharynx	1 (2.2%)	0		2 (5.4%)	1 (4.5%)	
Histological grading			1.00			0.132
G1	4 (12.5%)	2 (13.3%)		0 (0.0%)	1 (8.3%)	
G2	23 (71.9%)	11 (73.3%)		12 (54.5%)	9 (75.0%)	
G3	5 (15.6%)	2 (13.3%)		10 (45.5%)	2 (16.7%)	
			1.00			0.140
G1-2	27 (84.4%)	13 (86.7%)		12 (54.5%)	10 (83.3%)	
G3	5 (15.6%)	2 (13.3%)		10 (45.5%)	2 (16.7%)	
Tumor depth (T)			0.435			0.268
T1	7 (15.2%)	1 (4.5%)		5 (13.5%)	0 (0.0%)	
T2	16 (34.8%)	8 (36.4%)		12 (32.4%)	6 (27.3%)	
T3	12 (26.1%)	10 (45.5%)		13 (35.1%)	9 (40.9%)	

T4	10 (21.7%)	3 (13.6%)		7 (18.9%)	7 (31.8%)	
Tx	1 (2.2%)	0				
			0.450			0.179
T1-2	23 (50.0%)	9 (40.9%)		17 (45.9%)	6 (27.3%)	
T3-4	22 (47.8%)	13 (59.1%)		20 (54.1%)	16 (72.7%)	
Lymph node metastasis (N)			0.949			0.983
N0	14 (30.4%)	6 (27.3%)		4 (10.8%)	2 (9.1%)	
N1	10 (21.7%)	6 (27.3%)		6 (16.2%)	4 (18.2%)	
N2	16 (34.8%)	8 (36.4%)		21 (56.8%)	12 (54.5%)	
N3	6 (13.0%)	2 (9.1%)		5 (13.5%)	4 (18.2%)	
Nx				1 (2.7%)	0 (0.0%)	
			1.00			1.00
N0-1	24 (52.2%)	12 (54.5%)		10 (27.8%)	6 (27.3%)	
N2-3	22 (47.8%)	10 (45.5%)		26 (72.2%)	16 (72.7%)	
Treatment duration [days]	44.2±8.47 (6-60)	42.6±10.13 (6-56)	0.439	49.1±12.06 (36-114)	47.5±5.09 (38-56)	0.967
General treatment regimen			0.421			0.702
RT	18 (39.1%)	6 (27.3%)		5 (13.5%)	2 (9.1%)	
CRT	28 (60.9%)	16 (72.7%)		32 (86.5%)	20 (90.9%)	
Initial BMI [kg/m^2]	26.1±4.99 (17.2-37.6)	25.0±4.02 (18.3-32.4)	0.391	28.7±4.38 (21.8-40.4)	27.2±4.10 (1802-35.3)	0.249
BMI groups			0.590			0.373
<18.5	2 (4.3%)	2 (9.1%)		0 (0.0%)	1 (4.5%)	
≥18.5	44 (95.7%)	20 (90.9%)		37 (100%)	21 (95.5%)	
Smoking			1.00			0.255
Yes	31 (67.4%)	15 (68.2%)		15 (40.5%)	5 (22.7%)	
No	15 (32.6%)	7 (31.8%)		22 (59.5%)	17 (77.3%)	
Alcohol abuse			0.546			
Yes	3 (6.5%)	0		0 (0.0%)	0 (0.0%)	
No	43 (93.5%)	22 (100%)		37 (100%)	22 (100%)	

M, male; F, female; BMI, body mass index.

Table S4. Comparison of low and high monocyte/lymphocyte ratio (MLR) groups according to selected clinicopathological factors in HPV(-) and HPV(+) patients.

	HPV-			HPV+		
	**MLR < 0.36**	**MLR ≥ 0.36**	**p**	**MLR < 0.31**	**MLR ≥ 0.31**	**p**
Age [years]	60.7±6.52 (50-79)	61.1±8.80 (37-74)	0.497	61.3±9,44 (39-80)	58.8±10,06 (30-78)	0.475
Gender			0.154			**0.014**
M	27 (67.5%)	24 (85.7%)		18 (48.6%)	18 (81.8%)	
F	13 (32.5%)	4 (14.3%)		19 (51.4%)	4 (18.2%)	
General location			1.00			0.334
Tonsil	26 (65.0%)	18 (64.3%)		31 (83.8%)	16 (72.7%)	
Others	14 (35.0%)	10 (35.7%)		6 (16.2%)	6 (27.3%)	
Detailed location			0.925			0.103
1. tonsil	26 (65.0%)	18 (64.3%)		31 (83.8%)	16 (72.7%)	
2. palate	5 (12.5%)	5 (17.9%)		0 (0.0%)	0 (0.0%)	
3. root of the tongue	8 (20.0%)	5 (17.9%)		3 (8.1%)	6 (27.3%)	

4. other oropharynx	1 (2.5%)	0 (0.0%)		3 (8.1%)	0 (0.0%)	
Histological grading			**0.026**			1.00
G1	2 (7.1%)	4 (21.1%)		1 (5.3%)	0 (0.0%)	
G2	19 (67.9%)	15 (78.9%)		11 (57.9%)	10 (66.7%)	
G3	7 (25.0%)	0 (0.0%)		7 (36.8%)	5 (33.3%)	
			**0.032**			1.00
G1-2	21 (75.0%)	19 (100%)		12 (63.2%)	10 (66.7%)	
G3	7 (25.0%)	0 (0.0%)		7 (36.8%)	5 (33.3%)	
Tumor depth (T)			0.727			0.919
T1	6 (15.0%)	2 (7.1%)		4 (10.8%)	1 (4.5%)	
T2	14 (35.0%)	10 (35.7%)		11 (29.7%)	7 (31.8%)	
T3	12 (30.0%)	10 (35.7%)		13 (35.1%)	9 (40.9%)	
T4	8 (20.0%)	5 (17.9%)		9 (24.3%)	5 (22.7%)	
Tx	0 (0.0%)	1 (3.6%)				
			0.626			0.789
T1-2	20 (50.0%)	12 (42.8%)		15 (40.5%)	8 (36,4%)	
T3-4	20 (50.0%)	16 (57.2%)		22 (59,5%)	14 (63.6%)	
Lymph node metastasis (N)			0.342			0.153
N0	10 (25.0%)	10 (35.7%)		5 (13.5%)	1 (4.5%)	
N1	9 (22.5%)	7 (25.0%)		5 (13.5%)	5 (22.7%)	
N2	14 (35.0%)	10 (35.7%)		19 (51.4%)	14 (63.6%)	
N3	7 (17.5%)	1 (3.6%)		8 (21.6%)	1 (4.5%)	
Nx	0 (0.0%)	0 (0.0%)		0 (0.0%)	1 (4.5%)	
			0.330			1.00
N0-1	19 (47.5%)	17 (60.7%)		10 (27.0%)	6 (28,6%)	
N2-3	21 (52.5%)	11 (39.3%)		27 (73.0%)	15 (71,4%)	
Treatment duration [days]	44.5±6.81 (26-60)	42.4±11.41 (6-56)	0.944	47.1±4,83 (36-54)	51.0±15,20 (36-114)	0.452
General treatment regimen			0.613			0.702
RT	13 (32.5%)	11 (39.3%)		5 (13.5%)	2 (9.1%)	
CRT	27 (67.5%)	17 (60.7%)		32 (86.5%)	20 (90.9%)	
Initial BMI [kg/m^2]	26.7±4.78 (17.2-37.6)	24.4±4.33 (18.3-37.1)	0.029	28.3±4,76 (18,2-40,4)	27.8±3,47 (22,2-35,3)	0.772
BMI groups			1.00			1.00
<18.5	2 (5.0%)	2 (7.1%)		1 (2.7%)	0 (0.0%)	
≥18.5	38 (95.0%)	26 (92.9%)		36 (97.3%)	22 (100%)	
Smoking			0.793			0.571
Yes	28 (70.0%)	18 (64.3%)		14 (37.8%)	6 (27.3%)	
No	12 (30.0%)	10 (35.7%)		23 (62.2%)	16 (72.7%)	
Alcohol abuse			0.564			
Yes	1 (2.5%)	2 (7.1%)		0 (0.0%)	0 (0.0%)	
No	39 (97.5%)	26 (92.9%)		37 (100%)	22 (100%)	

M, male; F, female; BMI, body mass index.

Table S5. Comparison of low and high platelet/lymphocyte (PLR) groups according to selected clinicopathological factors in HPV(-) and HPV(+) patients.

	**HPV-**			**HPV+**		
	**PLR < 152.25**	**PLR ≥ 152.25**	**p**	**PLR < 145.09**	**PLR ≥ 145.09**	**p**

Age [years]	60.8±7.12 (47-79)	61.0±8.27 (37-74)	0.476	61.5±10.1 (30-80)	58.2±8.7 (39-75)	0.166
Gender			0.770			0.265
M	32 (72.7%)	19 (79.2%)		26 (66.7%)	10 (50.0%)	
F	12 (27.3%)	5 (20.8%)		13 (33.3%)	10 (50.0%)	
General location			0.070			0.305
Tonsil	32 (72.7%)	12 (50.0%)		33 (84.6%)	14 (70.0%)	
Others	12 (27.3%)	12 (50.0%)		6 (%)	6 (30.0%)	
Detailed location			0.174			0.322
1. tonsil	32 (72.7%)	12 (50.0%)		33 (84.6%)	14 (70.0%)	
2. palate	5 (11.4%)	5 (20.8%)				
3. root of the tongue	6 (13.6%)	7 (29.2%)		4 (10.3%)	5 (25.0%)	
4. other oropharynx	1 (2.3%)	0 (0.0%)		2 (5.1%)	1 (5.0%)	
Histological grading			0.788			0.309
G1	4 (12.5%)	2 (13.3%)		1 (4.3%)	0 (0%)	
G2	24 (75.0%)	10 (66.7%)		12 (52.2%)	9 (81.8%)	
G3	4 (12.5%)	3 (20.0%)		10 (43.5%)	2 (18.2%)	
			0.664			0.252
G1-2	28 (87.5%)	12 (80.0%)		13 (56.5%)	9 (81.8%)	
G3	4 (12.5%)	3 (20.0%)		10 (43.5%)	2 (18.2%)	
Tumor depth (T)			0.584			0.512
T1	7 (15.9%)	1 (4.2%)		4 (10.3%)	1 (5.0%)	
T2	14 (31.8%)	10 (41.7%)		14 (35.9%)	4 (20.0%)	
T3	13 (29.5%)	9 (37.5%)		13 (33.3%)	9 (45.0%)	
T4	9 (20.5%)	4 (16.7%)		8 (20.5%)	6 (30.0%)	
Tx	1 (2.3%)	0 (0.0%)				
			1.00			0.161
T1-2	21 (48.8%)	11 (45.8%)		18 (46.2%)	5 (25.0%)	
T3-4	22 (51.2%)	13 (54.2%)		21 (53.8%)	15 (75.0%)	
Lymph node metastasis (N)			0.967			0.981
N0	13 (29.5%)	7 (29.2%)		4 (10.3%)	2 (10.0%)	
N1	10 (22.7%)	6 (25.0%)		6 (15.4%)	4 (20.0%)	
N2	15 (34.1%)	9 (37.5%)		22 (56.4%)	11 (55.0%)	
N3	6 (13.6%)	2 (8.3%)		6 (15.4%)	3 (15.0%)	
Nx				1 (2.6%)	0 (%)	
						0.767
N0-1	23 (52.3%)	13 (54.2%)	1.00	10 (26.3%)	6 (30.0%)	
N2-3	21 (47.7%)	11 (45.8%)		28 (73.7%)	14 (70.0%)	
Treatment duration [days]	44.1±8.68 (6-60)	42.8±9.67 (6-52)	0.652	48.9±12.2 (36-114)	47.9±3.9 (39-56)	0.987
General treatment regimen			1.00			0.083
RT	16 (36.4%)	8 (33.3%)		7 (17.9%)	0 (%)	
CRT	28 (63.6%)	16 (66.7%)		32 (82.1%)	20 (100%)	
Initial BMI [kg/m^2]	26.4±4.43 (17.6-37.1)	24.6±5.04 (17.2-37.6)	0.087	28.7±4.8 (18.2-40.4)	26.8±2.8 (23.4-33.3)	0.094
BMI groups			0.122			1.00
<18.5	1 (2.3%)	3 (12.5%)		1 (2.6%)	0	
≥18.5	43 (97.7%)	21 (87.5%)		38 (97.4%)	20 (100%)	
Smoking			1.00			0.042
Yes	30 (68.2%)	16 (66.7%)		17 (43.6%)	3 (15.0%)	
No	14 (31.8%)	8 (33.3%)		22 (56.4%)	17 (85.0%)	
Alcohol abuse			1.00			
Yes	2 (4.5%)	1 (4.2%)		0 (0.0%)	0 (0.0%)	

No	42 (95.5%)	23 (95.8%)		39 (100%)	20 (100%)	

M, male; F, female; BMI, body mass index.

Table S6. Comparison of low and high systemic immune inflammation (SII) groups according to selected clinicopathological factors in HPV(-) and HPV(+) patients.

	**HPV-**			**HPV+**		
	**SII < 707.36**	**SII ≥ 707.36**	**p**	**SII < 553.31**	**SII ≥ 553.31**	**p**
Age [years]	60.9±6.9 (50-79)	60.7±8.7 (37-74)	0.621	60.7±10.2 (30-80)	59.7±8.7 (45-76)	0.532
Gender			0.770			0.780
M	32 (72.7%)	19 (79.2%)		23 (59.0%)	13 (65.0%)	
F	12 (27.3%)	5 (20.8%)		16 (41.0%)	7 (35.0%)	
General location			0.070			0.305
Tonsil	32 (72.7%)	12 (50.0%)		33 (84.6%)	14 (70.0%)	
Others	12 (27.3%)	12 (50.0%)		6 (15.4%)	6 (30.0%)	
Detailed location			0.092			0.322
1. tonsil	32 (72.7%)	12 (50.0%)		33 (84.6%)	14 (70.0%)	
2. palate	6 (13.6%)	4 (16.7%)				
3. root of the tongue	5 (11.4%)	8 (33.3%)		4 (10.3%)	5 (25.0%)	
4. other oropharynx	1 (2.3%)	0 (0%)		2 (5.1%)	1 (5.0%)	
Histological grading			1.00			0.021
G1	4 (13.3%)	2 (11.8%)		0 (0%)	1 (9.1%)	
G2	21 (70.0%)	13 (76.5%)		12 (52.2%)	9 (81.8%)	
G3	5 (16.7%)	2 (11.8%)		11 (47.8%)	1 (9.1%)	
			1.00			0.053
G1-2	25 (83.3%)	15 (88.2%)		12 (52.2%)	10 (90.9%)	
G3	5 (16.7%)	2 (11.8%)		11 (47.8%)	1 (9.1%)	
Tumor depth (T)			0.662			0.112
T1	7 (15.9%)	1 (4.2%)		5 (12.8%)	0 (0%)	
T2	15 (34.1%)	9 (37.5%)		13 (33.3%)	5 (25.0%)	
T3	13 (29.5%)	9 (37.5%)		15 (38.5%)	7 (35.0%)	
T4	8 (18.2%)	5 (20.8%)		6 (15.4%)	8 (40.0%)	
Tx	1 (2.3%)	0 (0%)				
			0.611			0.161
T1-2	22 (51.2%)	10 (41.7%)		18 (46.2%)	5 (25.0%)	
T3-4	21 (48.8%)	14 (58.3%)		21 (53.8%)	15 (75.0%)	
Lymph node metastasis (N)			0.967			0.918
N0	14 (31.8%)	6 (25.0%)		5 (12.8%)	1 (5.0%)	
N1	10 (22.7%)	6 (25.0%)		6 (15.4%)	4 (20.0%(	
N2	15 (34.1%)	9 (37.5%)		21 (53.8%)	12 (60.0%)	
N3	5 (11.4%)	3 (12.5%)		6 (15.4%)	3 (15.0%)	
Nx				1 (2.6%)	0 (0%)	
			0.802			1.00
N0-1	24 (54.5%)	12 (50.0%)		11 (28.9%)	5 (25.0%)	
N2-3	20 (45.5%)	12 (50.0%)		27 (71.1%)	15 (75.0%)	
Treatment duration [days]	45.2±6.2 (20-60)	40.8±12.2 (6-56)	0.164	48.6±12.0 (36-114)	48.3±4.7 (39-56)	0.403
General treatment regimen			0.288			0.404
RT	18 (40.9%)	6 (25.0%)		6 (15.4%)	1 (5.0%)	
CRT	26 (59.1%)	18 (75.0%)		33 (%	19 (95.0%)	

Initial BMI [kg/m²]	26.6±4.8 (17.2-37.6)	24.1±4.1 (18.3-32.4)	**0.028**	28.4±4.6 (18.2-40.4)	27.4±3.6 (22.2-35.3)	0.366
BMI groups			0.610			1.00
<18.5	2 (4.5%)	2 (8.3%)		1 (2.6%)	0 (0%)	
≥18.5	42 (95.5%)	22 (91.7%)		38 (97.4%)	20 (100%)	
Smoking			0.282			**0.042**
Yes	32 (72.7%)	14 (58.3%)		17 (43.6%)	3 (15.0%)	
No	12 (27.3%)	10 (41.7%)		22 (56.4%)	17 (85.0%)	
Alcohol abuse			0.547			
Yes	3 (6.8%)	0 (0%)		0 (0%)	0 (0%)	
No	41 (93.2%)	24 (100%)		39 (100%)	20 (100%)	

M, male; F, female; BMI, body mass index.

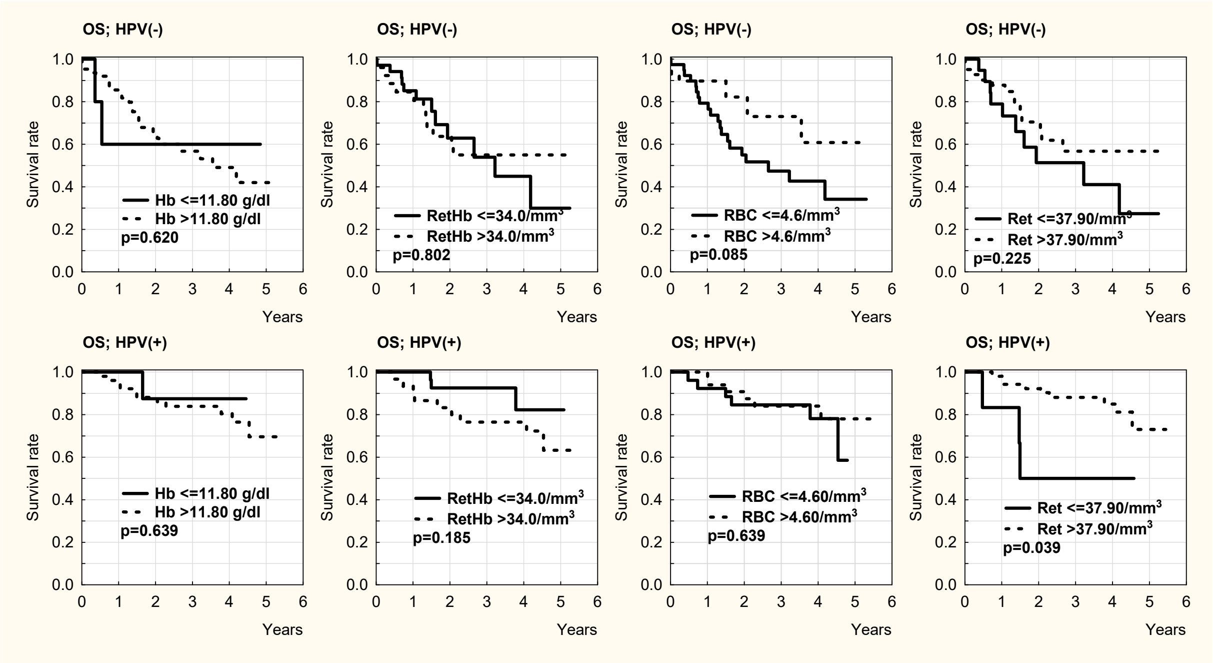

Figure S1. A. Overall survival (OS) in HPV- and HPV+ depending on hemoglobin (Hb), ret-hemoglobin (RetHb), reticulocyte count (Ret), red blood cell count (RBC).

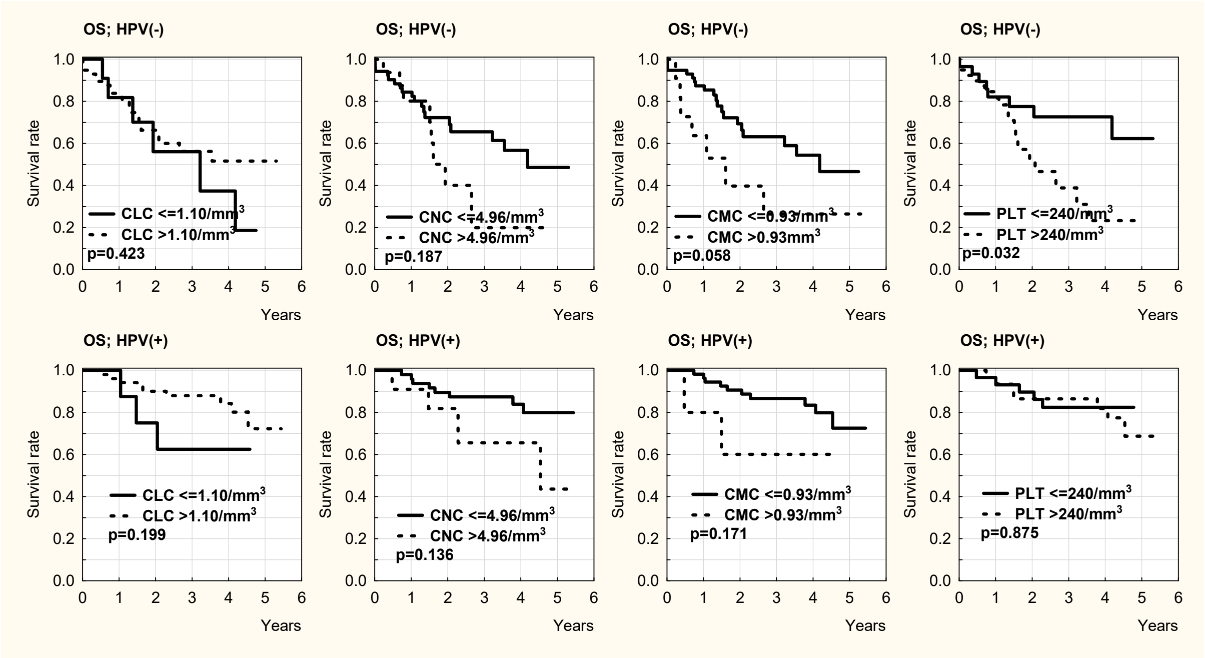

Figure S1. B. Overall survival (OS) in HPV- and HPV+ depending on circulating lymphocyte count (CLC), circulating neutrophil count (CNC), circulating monocyte count (CMC), platelet cell count (PLT).

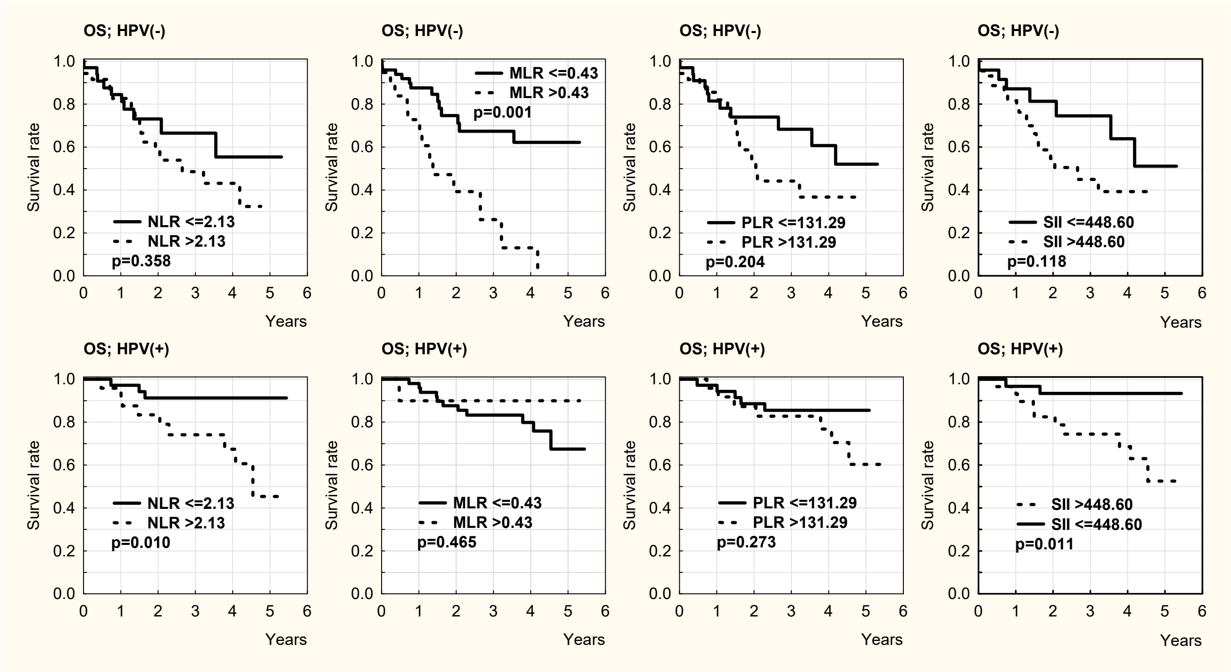

Figure S1. C. Overall survival (OS) in HPV- and HPV+ patients depending on neutrophil/lymphocyte ratio (NLR), platelet/lymphocyte ratio (PLR), and monocyte/lymphocyte ratio (MLR), and systemic immune inflammation index (SII).

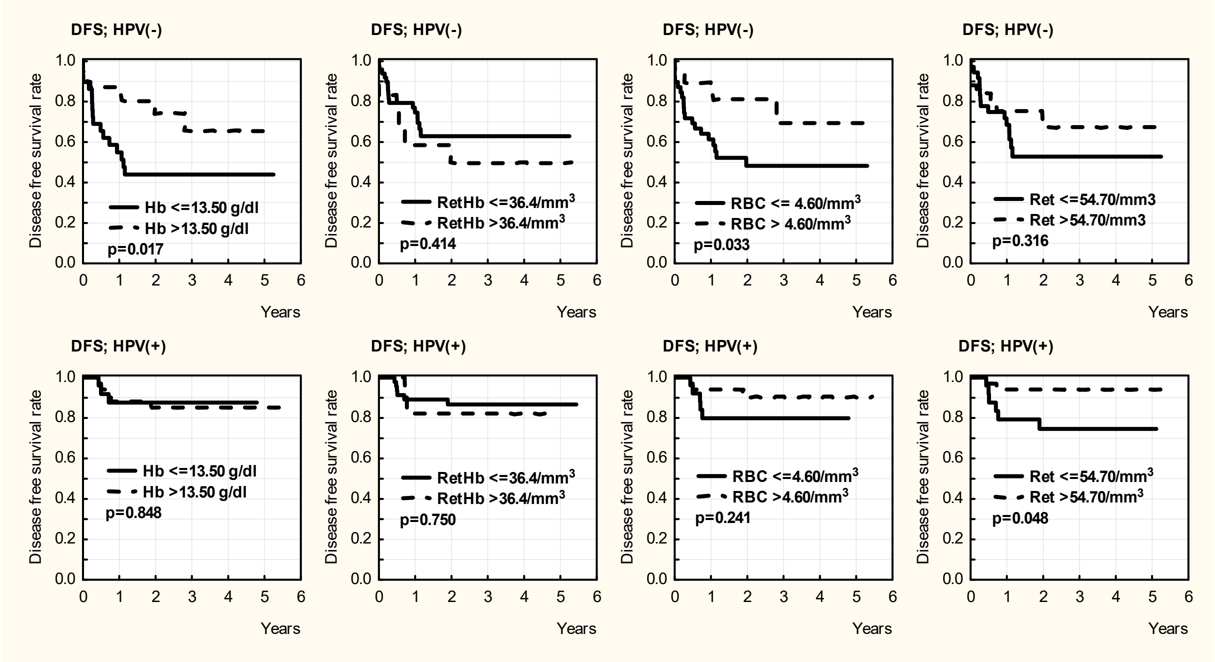

Figure S2. A. Disease-free survival (DFS) in HPV- and HPV+ patients depending on hemoglobin (Hb), ret-hemoglobin (RetHb), reticulocyte count (Ret), red blood cell count (RBC).

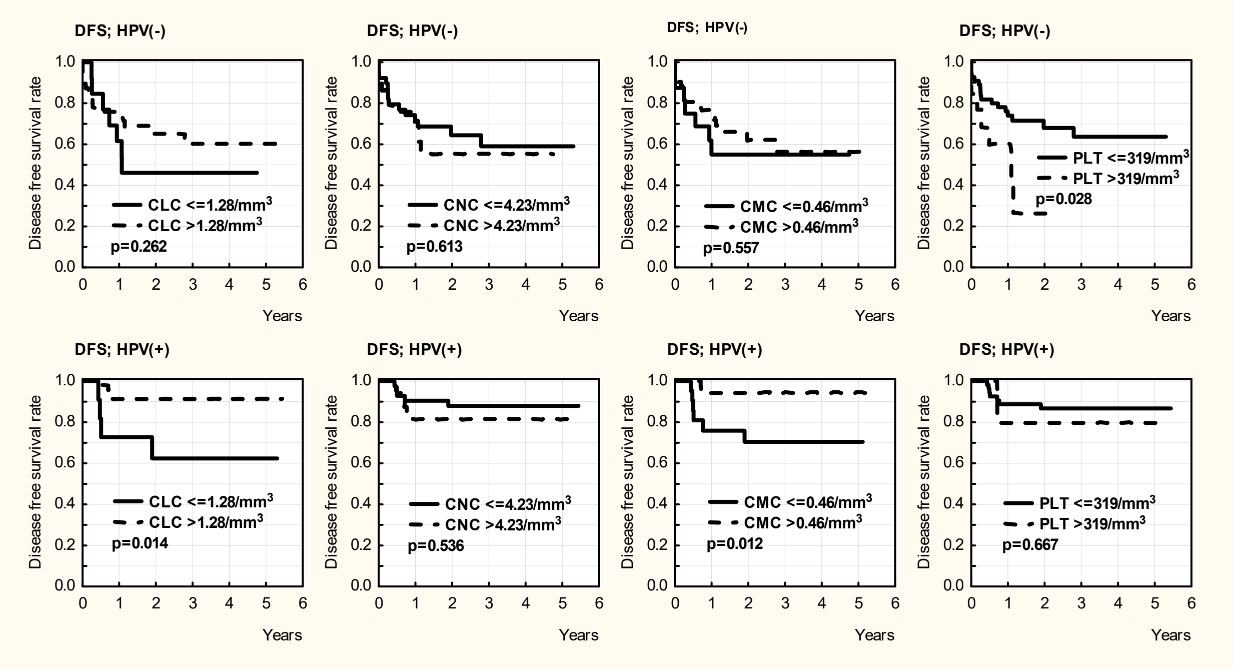

Figure S2. B. Disease-free survival (DFS) in HPV- and HPV+ patients depending on circulating lymphocyte count (CLC), circulating neutrophil count (CNC), circulating monocyte count (CMC), platelet cell count (PLT).

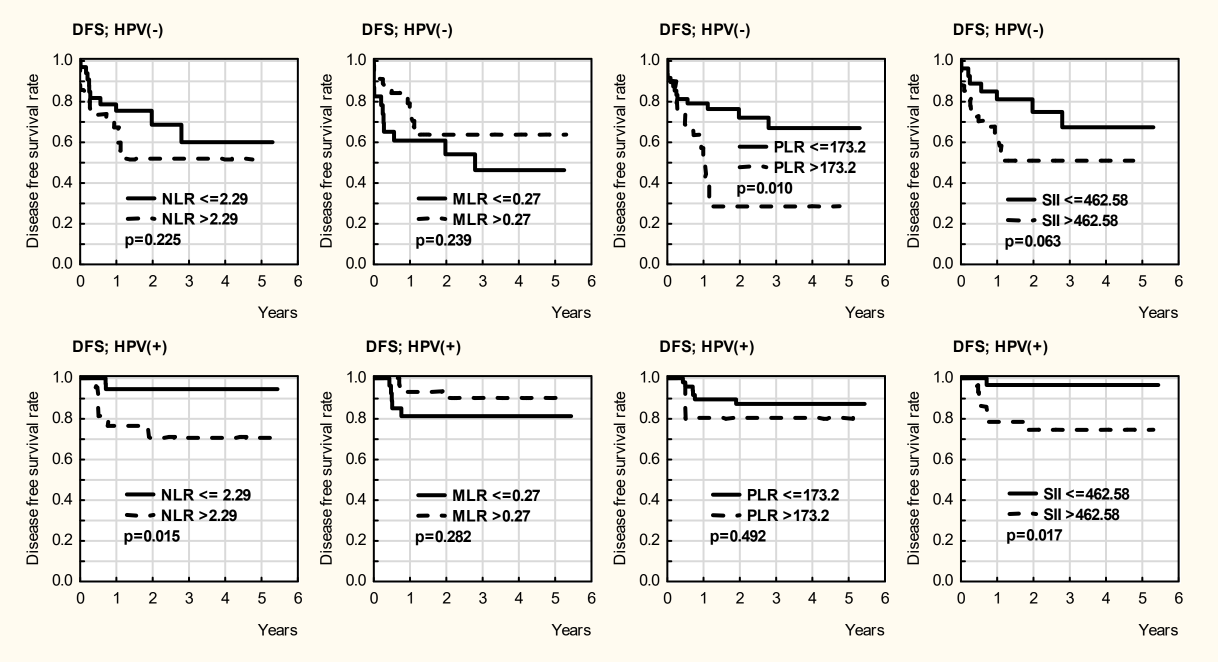

Figure S2. C. Disease-free survival (DFS) in HPV- and HPV+ patients depending on neutrophil/lymphocyte ratio (NLR), platelet/lymphocyte ratio (PLR), monocyte/lymphocyte ratio (MLR), and systemic immune inflammation index (SII).
